# Market-based measures and their impact on green shipping technologies

**DOI:** 10.1007/s13437-021-00258-8

**Published:** 2021-12-23

**Authors:** Daniel Metzger

**Affiliations:** 1 Hamburg School of Business Administration, Hamburg, Germany; 2 Helmut Schmidt University, Hamburg, Germany

**Keywords:** Green shipping, Market-based measures, Wind-assisted propulsion technologies, Shared savings, Ship finance, Fuzzy Pay-off Method

## Abstract

In the strategy on the reduction of greenhouse gas (GHG) emission of the International Maritime Organization (IMO), market-based measures (MBMs) are considered feasible mid-term measures. Thus, the relevance of MBMs for the shipping industry can be expected to grow in the future and, consequently, carbon and other GHG emissions will impact the investment appraisal for greening technologies. This paper illustrates the impact of carbon pricing on the valuation of greening technologies (especially wind-assisted propulsion technologies) and on the relevant decision-making. In this regard, the straightforward approach of a direct acquisition and installation of the respective technology is considered and compared against innovative financing models, such as shared savings. Hence, the Fuzzy Pay-Off Method (FPOM) is applied in order to visualize the risks and chances linked to MBMs. Due to the economic life of greening technologies, the results are already relevant for today’s investment appraisals, even though carbon pricing has not been enforced so far.

## Introduction

Looking at both the IMO objectives set out in its initial strategy on the reduction of greenhouse gas (GHG) emissions (IMO 2018) and the objectives set out in the Paris Agreement, the greening of the maritime industry is indispensable (Cames et al. 2015). However, the goal of cutting GHG emissions by a minimum of 50% against 2008 levels until 2050 (IMO 2018) becomes increasingly challenging when taking projected annual growth rates of 3.4% (2019–2024) (UNCTAD 2019) for seaborne trade into account (Chen et al. 2019).

In order to archive this goal, the IMO has formulated several short-, mid-, and long-term measures. Simplified, these measures can be categorized in three categories, which are linked to one another: technological measures, operational measures and market-based measures (MBMs). The technological measures include the development and on-board installation of greening technologies. Operational measures are measures that improve a ship’s operations (i.e. via route and speed optimizations). The third category, MBMs, is mainly about carbon/emission pricing. It is debated on introducing either a levy (also called tax or fee) on emitted GHG emissions or an emission trading system (ETS), which would sponsor emission savers and punish polluters.

Technological measures such as wind-assisted ship propulsion (WASP), innovative hull designs, alternative fuels and others play an important role in shipping’s decarbonization process, as outlined in many studies and papers (i.e. Bouman et al. (2017), Rehmatulla et al. (2017), Halim et al. (2018), Balcombe et al. (2019)). However, the implementation of technological measures correlates with the introduction of MBMs, as the pricing of emissions makes emission abatement technologies more attractive (Cheaitou and Cariou 2019; Fan and Huang 2019; Schinas and Metzger 2019a; Metzger and Schinas 2019; Schwartz et al. 2020).

This paper aims at illustrating the impact of emission pricing on the financing of greening technologies. Thereby, sensitivities in relation to pricing and timing (i.e. enforcement dates) are calculated. Further, the question whether MBMs are necessary to make investments in greening technologies (and especially WASP technologies) attractive should be answered. Therefore, a literature review is conducted first. Afterwards, the impact of carbon-prizing on a WASP technology investment is shown. In order to show the impact of the applied financing scheme on the valuation, not only a direct (plain vanilla) purchase of the technology, but also financing via shared savings is considered. The Fuzzy Pay-Off Method (FPOM), as developed by Collan et al. (2009), is used for the visualization of risks and opportunities linked to MBMs. In the final part, the results are discussed and recommendations are derived.

## Literature review

The IMO Assembly instructed the Marine Environment Protection Committee (MEPC) to evaluate technical, operational and market-based solutions for the reduction of GHG emissions in 2003 (IMO 2003). Over 6 years later, ten different MBM proposals were submitted. In the following years, some stakeholders withdrew, revised or consolidated their proposals. In addition, another proposal was added by Germany (2010). Simplified, the proposals can be categorized in either supporting an ETS or a levy system (also called tax or fee). It is to be mentioned that the proposal submitted by the International Union for the Conservation of Nature (IUCN) (2010), which considers a rebate for development countries, is to be assessed as an add-on to the other proposals. A comparison of the proposals is inter alia provided by Psaraftis (2012). However, even though an IMO-mandated expert group evaluated the proposals (IMO 2010), the discussion was suspended in May 2013 (Psaraftis and Woodall 2019).

In 2017, the international pressure on the shipping industry increased substantially, when the European Parliament intended to include shipping into the European Union (EU) onshore ETS from 2023 onwards. Even though the EU withdrew their plan, they keep monitoring the IMO and its progress towards the adoption of an MBM (Psaraftis and Woodall 2019). Finally, the initial IMO strategy on the reduction of GHG emissions (IMO 2018), which considers MBMs as medium-term measures, was adopted in 2018. Therefore, the MBM discussion will once again be on the IMO agenda, though with more international pressure than in the years 2003–2016. Prior to MEPC 75, Psaraftis and Kontovas (2021) analysed the status quo of the initial IMO strategy. They argue that the discussed measures may help to achieve the 2030 carbon emission reduction goals (i.e. 40% reduction against 2008 levels), but they are not sufficient for the 2050 long-term goal since there are no other mid- or long-term measures other than emission pricing on the table for now. This underlines the increasing importance of MBMs for the industry. Recently, Møller Mærsk called for a considerable carbon tax, which shows that (some) ship operators, who would need to forward the carbon price to their customers, are open for MBMs (Wittels 2021). Nevertheless, it needs to be mentioned that there are no final decisions in relation to carbon pricing so far. Technically, there still is a chance that carbon pricing will never be introduced.

At MEPC 75 (November 2020), with the introduction of the Energy Efficiency Ship Index (EEXI), the existing Energy Efficiency Design Index (EEDI) regulation has been extended to cover all existing ships. Further, the Carbon Intensity Indicator (CII) has been introduced for ships above 5,000 GT. The CII is an annual carbon efficiency ratio that will be rated from A to E, while the efficiency requirements will increase over time. A low rating (E in a single year or D in three consecutive years) triggers the requirement to implement a corrective action plan into the Ship Energy Efficiency Management Plan (SEEMP) (IMO 2020). The policies were adopted at MEPC 76 (June 2021) and come into force at 1 January 2023. Neither EEXI nor CII have a direct impact on the MBM discussion, though both measures will help to reach the 2030 emission reduction goals. At MEPC 76, a proposal for a levy of USD 100 per tonne of CO_2_ equivalent has been discussed. The proposal was submitted by the Marshall Islands and the Solomon Islands. The proposal will be considered by the respective working group when assessing measures to decrease GHG emissions.

Looking at current research in the field of MBMs, the research of Gu et al. (2019) illustrates that the considered ETS might not guarantee for short-term emission reductions. They find that other factors (i.e. charter rates) might be more important for decision-makers than emission allowances. However, a scenario with high allowances and a global enforcement of the ETS seems most promising in terms of emission reduction. Furthermore, Gu et al. (2019) assess the ETS as most effective in a low fuel price environment. According to ben Brahim et al. (2019), a price for carbon and equivalent emissions of EUR 350–450 per ton would be required to achieve carbon neutral Danish shipping by 2050. This price seems out of the scope, as it might double cargo transportation costs. However, ben Brahim et al. (2019) illustrate that, due to the minor share of transportation costs in a shipped good, the overall price of shipped goods increases by only 6–8% on average. In general, it can be said that the future prices per ton carbon emissions are uncertain and subject to future MEPC meetings. The International Monetary Fund (IMF) assesses a global cross-sector carbon price of USD 75 per ton as necessary to achieve the objectives set out in the Paris Agreement (Parry 2019). Another critical aspect that needs to be mentioned is the monitoring and reporting of emissions, as illustrated by Rony et al. (2019) and Psaraftis and Woodall (2019), which needs to be improved when thinking about global enforcement of emission regulation. Interestingly, the COVID 19 pandemic does not materially impact the relevance of “green shipping” for the industry as argued by Prokopenko and Miśkiewicz (2020).

Under the IMO GHG strategy, various technologies qualify as technological measures (IMO 2018). The majority of these technologies can be installed in parallel, so that the emission reduction potential adds up to some extent (though this is not a linear function), as Bouman et al. (2017) elaborate. The latter assess the emission reduction potential of 22 abatement options. According to their study, the highest reduction potentials can be observed at biofuels. Svanberg et al. (2018), who analyse bio methanol as emission abatement option, argue that the successive introduction of bio methanol comes with rather low barriers and challenges. Biofuel could be used as a complement to fossil fuel during a transition phase. Such a hybrid approach (i.e. using multiple fuels) is analysed by Sui et al. (2020), though they do not cover biofuels. ben Brahim et al. (2019), who analyse pathways for Denmark towards climate-neutral shipping, argue in favour of either methanol, hydrogen or ammonia. However, Prussi et al. (2021) show that, as of 2021, the required infrastructure for such alternative fuels has not been established. Prussi et al. (2021), ben Brahim et al. (2019) and Svanberg et al. (2018) advocate further research in the field of alternative fuels in order to overcome, i.e. infrastructure, safety and supply-related uncertainties.

Another abatement option with relatively high reduction potential is slow steaming/speed reduction, which is to be characterized as an operational measure. This abatement option is easy to apply, as no capital expenditures (CAPEX) are required. However, the topic is controversial in academia, as the environmental benefits might be paid with economic losses (Cheaitou and Cariou 2019). Furthermore, the competitiveness of sea transportation against rail, road and airborne transportation decreases when the transportation time increases. Thus, speed cannot be reduced infinitely (Kosmas and Acciaro 2017). Consequently, substantial speed reductions without other measures are insufficient options in the long run. Contrary, in the short run, substantial speed reductions are a cheap alternative to the CAPEX required for greening technologies, which might be hindering for the development of new emission abatement technologies that will be required in the long run. This is one of the reasons why Psaraftis (2019) advocates MBMs instead of speed limits when it comes to reducing GHG emissions. However, as the studies of Bouman et al. (2017), Halim et al. (2018), and Cheaitou and Cariou (2019) suggest, a mix of technological and operational measures (both supported by MBMs) seems to be the most efficient option to minimize GHG emissions. Thus, slow steaming cannot be declined per se. For instance, the route and speed optimization model, which is developed by Zhen et al. (2020), illustrates that well-considered speed and route controlling in emission control areas is promising from an economical point of view.

As early-stage technologies such as bio methanol will require more years of research and development, this paper considers Flettner Rotors, a WASP technology, in the numerical examples. Flettner Rotors seem to be an attractive emission abatement technology when compared to others (Rehmatulla et al. 2017). Further, when using the economics of a Flettner Rotor, the results can be compared with similar research papers such as Schinas and Metzger (2019b). It can be observed that WASP technologies are currently experiencing a renaissance (Chou et al. 2021). The latter analyse available studies on the savings potential of Flettner Rotors and other WASP technologies. Their findings support the fuel-saving assumptions used in the following paragraphs, although it must be said that the fuel-saving potential correlates with the respective routes. Bentin et al. (2016) argue that route optimization can increase the fuel savings by WASP technologies materially (i.e. double the savings). The impact of different routes is also assessed in Tillig and Ringsberg (2020).

According to Kim and Seo (2019), who analyse the Korean maritime industry, the CAPEX linked to emission abatement measures are the most significant decision criterion for shipping companies when it comes to compliance with emission regulation. Their results are supported by the findings of Tran et al. (2020). Halff et al. (2019) argue that the current IMO standards (i.e. regarding the sulphur cap) support those who comply with new standards at the very last moment. Contrary, ship operators, who invest earlier in measures required by future regulation than required, will not be compensated for their investment by governmental institutions or their customers (as it is illustrated by Schinas (2018)). The market’s current unwillingness to pay for an innovation premium (or greening premium) leads to several ideas on how to stimulate the decarbonization process. Some researchers such as Halff et al. (2019), Karslen et al. (2019) and Zis et al. (2019) propose external financial incentives (i.e. subsidies); others such as Schinas et al. (2018) propose financial support via export credit schemes, which substantially decrease the financing costs for green investments. An alternative or supplement to these is innovative financing methods for greening technologies such as shared savings (Schinas and Metzger 2019a). The latter decreases the initial cash commitment of the ship operator and shares the opportunities and risks linked to the investment between shipping company and the supplier or a financier. Thus, the model addresses problems such as the insufficient access to cash, which is required for purchasing a technology, and the uncertainty in regard to the technology’s performance. A comprehensive analysis of the split incentives between ship owners and operators when it comes to energy efficiency investments is provided in Rehmatulla and Smith (2020). They have surveyed 275 ship owners and their findings indicate that ship owners, who are less dependent on fuel prices (i.e. via time charter agreements), have a higher energy efficiency technology implementation rate than ship owners with spot charter agreements (i.e. higher fuel price exposure), which represents a disconnect between theoretical hypothesis and reality.

In order to determine the impact of carbon pricing on the financing of greening technologies, the net present value (NPV) expression for greening technologies of Schinas and Metzger (2019a) is used in the following. This NPV expression already considers carbon pricing, which depends on MBMs. In order to compare the plain vanilla purchase of a technology with the shared savings financing scheme, the NPV of a technology acquired via a purchase is referred to as NPV_Purchase_. The NPV of a technology investment financed via the pay-as-you-save (PAYS) scheme, which is the shared savings scheme developed by Schinas and Metzger (2019a), is referred to as NPV_PAYS−Ship_ for the shipping company and NPV_PAYS−Fin_ for the financier. Assuming an equal discount factor for the cash flows, PAYS is to be expressed as:

1 $${\mathrm{NPV}}_{\mathrm{PAYS}-\mathrm{Ship}}\left(\lambda ,\tau ,{\tau }^{'}\right)+{\mathrm{NPV}}_{\mathrm{PAYS}-\mathrm{Fin}}\left(\lambda ,{\tau }^{'}\right)={\mathrm{NPV}}_{\mathrm{Purchase}}$$

where $\lambda$ is the shared savings fraction of the financier/supplier {0, 1}; $\tau$ is the expected lifetime of the technology; $\tau'$ is the shared savings contract duration {$\tau'$  $\le$ $\tau$}.

For a detailed definition of each parameter, see Schinas and Metzger (2019a). The main characteristics of PAYS are the participation of the supplier (or another third party) in the initial acquisition costs for a technology and her following participation in the savings achieved by the same technology during a fixed period ($\tau'$). For the scope of this paper, the presented elaboration on the PAYS model is sufficient. However, in the later calculations, all the parameters of the original model are considered.

The FPOM is helpful for decision-makers when comparing different greening technologies. It boils down NPV, risks and opportunities into a single number, which makes greening technologies—with different risk profiles—comparable to each other. It has been developed by Collan et al. (2009) and was applied in various scenarios ever since. In 2019, it was introduced to the maritime space (Metzger and Schinas 2019). Since then, the methodology has been acknowledged by other researchers in the field of green shipping (i.e. Ma et al. (2021) and Wang et al. (2021)). Due to the many parameters, there may be not one but many solutions when evaluating a greening technology. So, it can be argued that a fuzzy methodology is more suitable for such mathematical problems than crisp methods. Therefore, the FPOM is applied in the following numerical examples. Collan et al. (2009) developed the FPOM originally as an alternative to established real option valuation methods. However, the method can also be used to compare the risk profile of different investment opportunities, as it is illustrated by Metzger and Schinas (2019) in the maritime context. Collan et al. (2009) use triangular fuzzy numbers in order to derive an adjusted NPV of the underlaying asset (referred to as fuzzy NPV (FNPV)). The triangle’s corner points are defined by the base-, worst-, and best-case scenarios of the investment, while the height is always 1. Kozlova et al. (2016) show that the results of the method are comparable to the results of similar real option approaches such as Mathews et al. (2007). The method can be visualized, which makes it user friendly and intuitive, as it will be shown in the following sections.

The possibilistic mean, as defined by Carlsson and Fullér (2001), represents the value of the fuzzy number. When evaluating real options, the possibilistic mean can lead to theoretically incorrect results (i.e. negative option values), as illustrated by Borges et al. (2018). Therefore, Borges et al. (2018) enhance the model by replacing the possibilistic mean with the centre of gravity (CoG), resulting in the CoG-FPOM. However, when comparing investment opportunities, as it will be done in the following section, this theoretical incorrectness does not matter, as long as the methods are not mixed (i.e. one opportunity is evaluated by the FPOM and another by the CoG-FPOM). Both methods are compared by Metzger and Schinas (2019) and the results indicate that using only one of both methods is sufficient for decision-makers, as both methods come to similar conclusions. Therefore, the original FPOM is considered in the following paragraphs.

**Fig. 1:**
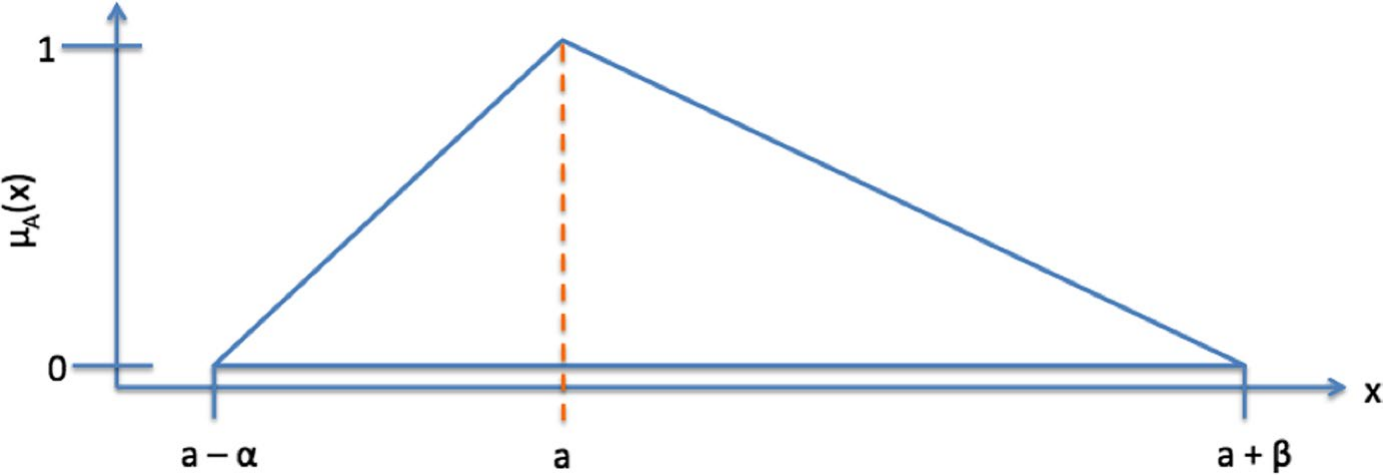
Triangular fuzzy number A (Metzger and Schinas 2019; similar to Borges et al. 2018)

A triangular fuzzy number $\mathrm{A}(\mathrm{a},\mathrm{\alpha },\upbeta$) is portrayed in Fig. 1 (from Metzger and Schinas (2019); similar to Borges et al. (2018)). A fuzzy number A is defined as:

2 $${\mu }_{A}(x)=\left\{\begin{array}{c}1-\frac{a-x}{\alpha } for a-\alpha \le x\le a\\ 1-\frac{x-a}{\beta } for a\le x\le a+\beta \\ 0 \mathrm{\:otherwise}\end{array}\right.$$

where ${\mu }_{A}(x)$ is the membership function; $a$ is the peak; $\alpha$ is the left width; $\beta$ is the right width.

In the FPOM, the corner points are defined as follows (Collan et al. 2009):

3 $$a={NPV}_{\mathrm{base}}=\mathrm{NPV\:of\:the\:base}-\mathrm{case\:scenario}$$

4 $$a-\alpha ={NPV}_{\mathrm{worst}}=\mathrm{NPV\:of\:the\:worst}-\mathrm{case\:scenario}$$

5 $$a+\beta ={NPV}_{\mathrm{best}}=\mathrm{NPV\:of\:the\:best}-\mathrm{case\:scenario}$$

**Fig. 2:**
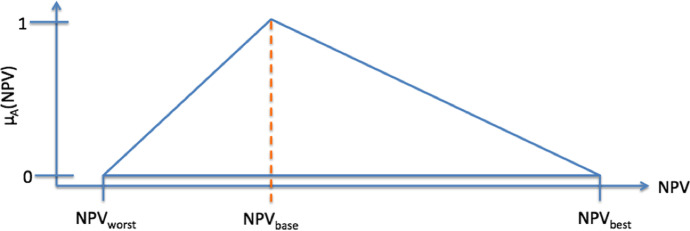
Investment as triangular fuzzy number A (Metzger and Schinas 2019)

The fuzzy number A (Fig. 2) represents the investment. The membership of 1 (${\mu }_{A}(NPV)=1$), which indicates the maximum possibility within a fuzzy set, is assigned to the most probable scenario (${NPV}_{\mathrm{base}}$).

The FNPV is defined as the possibilistic mean E(A +) times the ratio of the positive area to the whole area of the fuzzy number (Collan et al. 2009):

6 $$\mathrm{FNPV}=E({A}_{+})\times \frac{{\int }_{0}^{\infty }{\mu }_{A}(\mathrm{NPV})\mathrm{dNPV}}{{\int }_{-\infty }^{\infty }{\mu }_{A}(\mathrm{NPV})\mathrm{dNPV}}$$

For the calculation of E(A +), see Carlsson and Fullér (2001). A numerical example in the maritime context is provided in Metzger and Schinas (2019).

## Assessing the impact of MBMs on greening technologies

This section aims at illustrating how MBMs impact the valuation of a greening technology. Therefore, several carbon pricing scenarios are calculated for the same technology. Each scenario is calculated for a plain vanilla purchase of the technology and the shared savings scheme described in the literature review. Finally, the risks and opportunities linked to MBMs are visualized by applying the FPOM. In order to make the scenarios more tangible, the following calculations are made for the Flettner Rotor technology, which is one of the most prominent technologies at the moment.

First, the characteristics of the ship need to be stated in order to determine its fuel consumption. It is assumed that the technology (i.e. two Flettner Rotors) is mounted on a general cargo ship, which operates on about 300 sea days per year:

- The weighted average cost of capital (WACC) of the shipping company is 9.0%.
- The IFO380 demand of the ship per sea day is 45t, which could be the consumption profile of a medium- to large-sized cargo or container ship. The following scenarios also consider very low sulphur fuel oil (VLSFO). In order to compare IFO380 and VLSFO, it is assumed that the same ship would use c. 40.7t of very low sulphur fuel oil (VLSFO) instead of 45t of IFO380.
- The average price per ton IFO380 is USD 350, which equals 3.116t CO_2_.
- The average price per ton VLSFO is 25% more expensive than IFO380 (USD 437.5), which equals 3.188t CO_2_ (Comer and Osipova 2021).

The price of IFO380 reflects the top 20 ports’ average price for IFO380 from October 2020 to October 2021. It is assumed that the fuel price increases by 3% p.a., which roughly reflects the oil price development of the last 5 years prior to the COVID 19 pandemic. The following scenarios also consider very-low-sulphur fuel oil (VLSFO) since it is widely used by the industry. It is assumed that VLSFO is 25% more expensive than IFO380, which represents the average price differential between October 2020 and 2021. Further, it is assumed that VLSFO is c. 4.5% more efficient than IFO380 while having a carbon factor of 3.188t CO_2_/t (Comer and Osipova 2021) and that the ship using IFO380 burns 5% more fuel for a scrubber. In total, VLSFO makes the ship 9.5% more efficient. This way, the results for VLSFO and IFO380 are made as comparable as possible.

It needs to be noted that when using other fuels, consumption profile, relative fuel savings achieved by the green technology, carbon conversion factor, and fuel prices change. Especially the fuel price differentials to other fuels are difficult to predict (see Schinas and Stefanakos (2013), who assess the price differential between low- and high-sulphur fuel with statistical methods).

For the Flettner Rotors, the following assumptions are made:

- The Flettner Rotors cost USD 2,000,000 and can be used on 60% of all sea days;
- The technology can be used for at least 10 years;
- Maintenance costs for year 1: USD 10,000 with an annual exponential increase of ${e}^{0.15}$;
- It saves about 13% of fuel consumption per sea day, which is an assumption also used in similar research (i.e. Schinas and Metzger (2019b)). The assumption also fits into the fuel saving analysis conducted by Chou et al. (2021) and an experiment conducted by DNV GL and University of Applied Sciences Emden/Leer (DNV GL 2019).

When using the PAYS model, two more parameters need to be defined:

- The ship owner pays 25% of the price (USD 500,000), while the rest is financed by the counterparty;
- 90% of the savings achieved in the first 6 years belong to the financier/supplier.

**Fig. 3:**
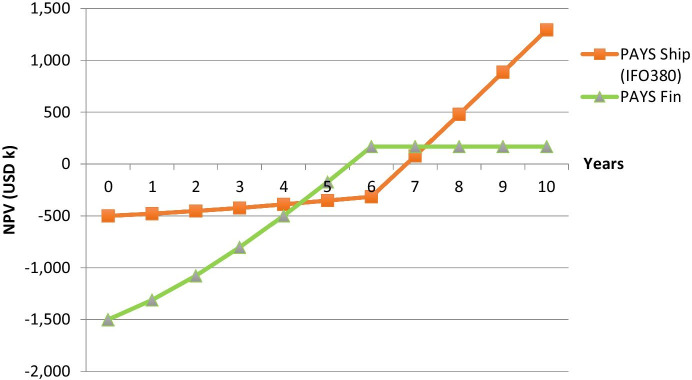
Pay-as-You-Save (PAYS) NPV for Flettner Rotors

Figure 3 visualizes how the shared savings model works for ship owner and the counterparty (supplier or financier), when applying the same discount rate for both. For the purpose of this graph, a value of USD 20 per ton of saved carbon emissions is assumed.

**Fig. 4:**
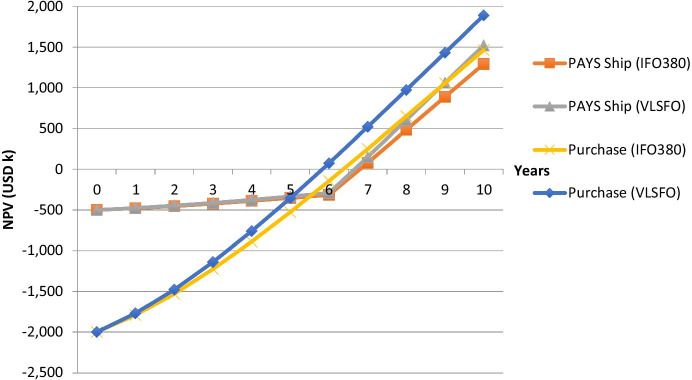
Purchase and PAYS profiles for Flettner Rotors

The graph shows the development of the NPV for both parties. After the sharing period, all savings are credited to the shipping company. The payoff profile for VLSFO looks similar and can be seen in Fig. 4, which compares PAYS (for the shipping company) and the traditional purchase of the technology. It can be seen that the NPV of the technology increases for the shipping company if it pays for the technology upfront. The delta between the Purchase and PAYS scenarios can be interpreted as a risk premium. Further, it can be seen that the NPV when using VLSFO is higher than the NPV when using IFO380. This is driven by the higher fuel savings when using VLSFO. The 25% higher price for VLSFO is not fully compensated by the efficiency improvement (assumed to be 9.5%).

**Fig. 5:**
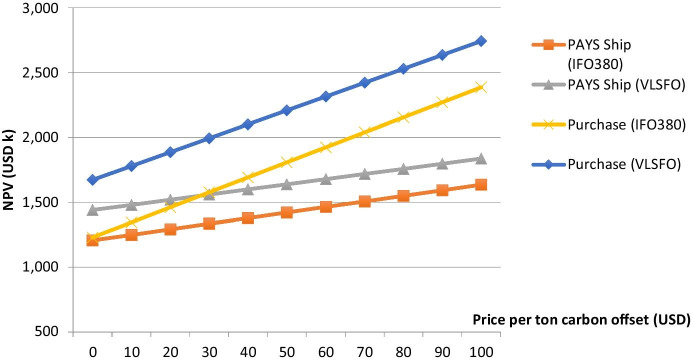
Carbon pricing scenarios for Flettner Rotors

Taking into account IMF’s estimation of a necessary price of USD 75 per ton of carbon emissions (across all sectors) (Parry 2019), USD 20 per ton is considered a conservative base-case in the following. However, Fig. 5 shows the impact of prices reaching from USD 0–100 per ton carbon equivalent. The upper end of the range (USD 100 per ton of carbon emissions) reflects the levy that has been proposed by the Marshall Islands and Solomon Islands in 2021. It is to be mentioned that no matter which MBM (levy or ETS) will be enforced in the future, the result will be that carbon emissions are priced with a certain value. Therefore, it is not differentiated between the levy and the ETS in the following.

When considering the critical carbon prices for the Danish case of ben Brahim et al. (2019) (at least EUR 350 per ton carbon emissions), NPV_PAYS−Ship_ equals USD 2,843 k and NPV_Purchase_ equals USD 5,643 k (considering the IFO380 scenario and an exchange rate of 1.09 EUR/USD). Looking at Figs. 4 and 5, it can be seen how PAYS limits the downside risks but also upside potentials for the shipping company.

**Fig. 6:**
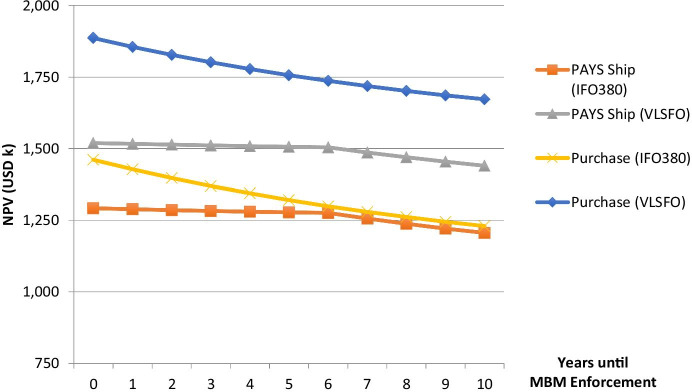
MBM enforcement scenarios for Flettner Rotors

In addition to the price uncertainties, it is not clear when the legislation for the respective MBM will be adopted and enforced. However, especially for the coming years, the enforcement date is of importance. Therefore, Fig. 6 shows the impact of the MBM enforcement date on the overall valuation of the technology. Thereby, the base-case value per emitted ton of carbon emissions (USD 20) is considered.

Figure 6 shows that the PAYS model protects the shipping company to some extent from a delay in the MBM enforcement process. However, it needs to be mentioned that material uncertainties would be reflected in the counterparty’s risk premium. Therefore, it might be an option to exclude the carbon offset revenue from the shared savings (see Schinas and Metzger (2019a)) when the MBM enforcement date is too uncertain for the counterparty’s risk appetite. Nevertheless, Figs. 5 and 6 illustrate that the described WASP technology investment would even pay-off without any carbon allowances.

Finally, the FPOM, as it is introduced in the literature review, is used to show the risks and opportunities of both financing options and make them comparable to other greening technologies. Therefore, worst-, base- and best-cases need to be defined. For the base-case scenario, a carbon prize of USD 20, which will be enforced after 5 years of operations, is considered. This time frame is reasonable when taking into account the IMO GHG strategy (IMO 2018). The base-case scenario results in an IRR of 28.4% for the shipping company when applying PAYS and an IRR of 18.9% when acquiring the technology the traditional way (IFO380 scenario).

**Fig. 7:**
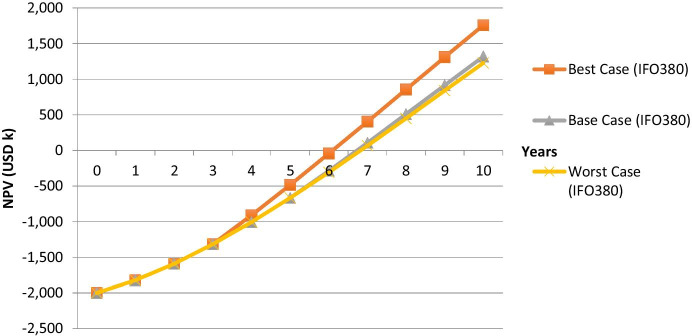
MBM scenarios for the Purchase option (IFO380)

**Fig. 8:**
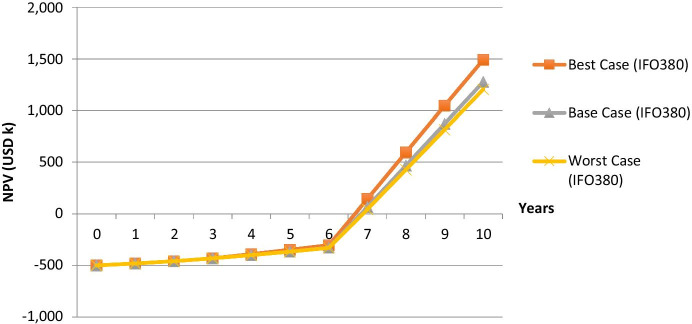
MBM scenarios for the PAYS option

For the worst-case scenario, it is considered that the MBM is not introduced within the useful life of the technology. Thus, the carbon offset revenue is zero. In the best-case scenarios, a price of USD 75 per ton carbon emissions (IMF scenario (Parry 2019)) is enforced after 3 years of operations. Figures 7 and 8 visualize the described scenarios when using IFO380 (the VLSFO scenarios are shown in Appendix 1).

**Fig. 9:**
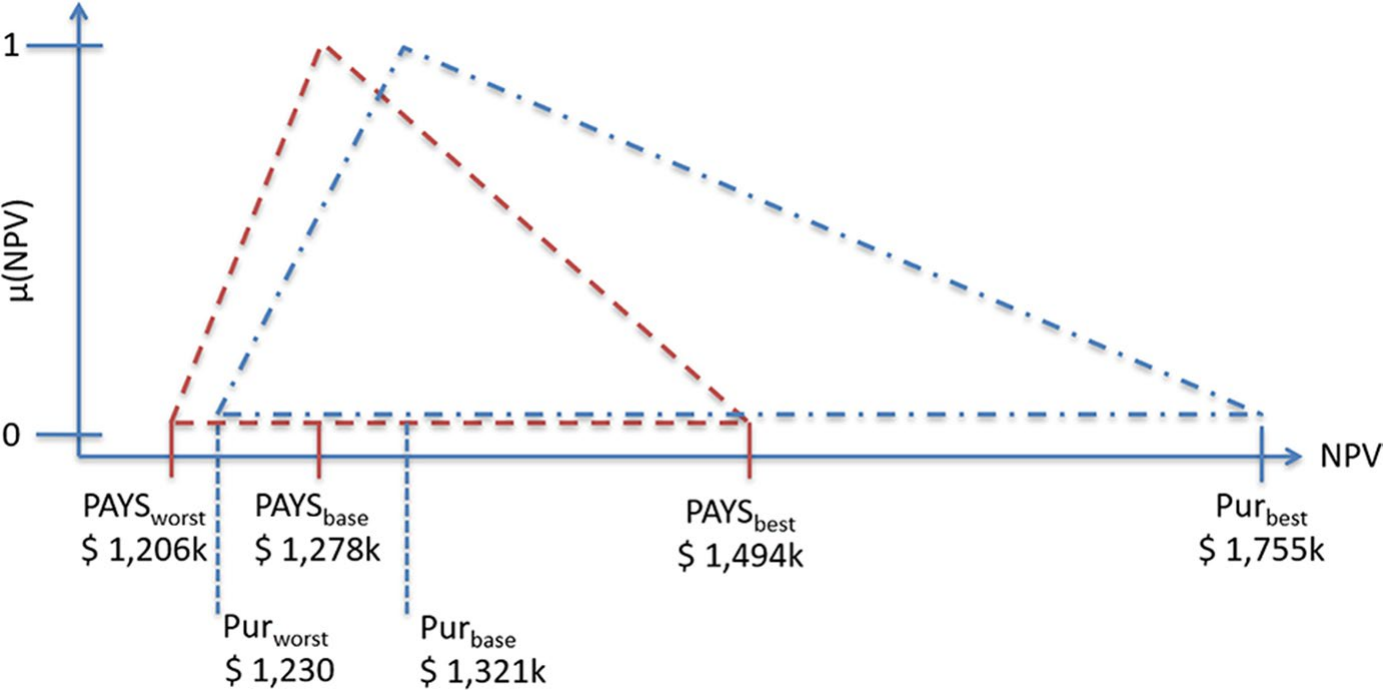
Payoff distributions (PAYS and Purchase) under consideration of MBM scenarios (IFO380 case)

Figure 9 translates the abovementioned scenarios into fuzzy numbers (fuzzy pay-off distributions) for the IFO380 case (the pay-off profile for the VLSFO case does look similar). The triangles, which are simplified pay-off distributions, should include all of the possible NPV scenarios, for the described case (i.e. different carbon prices and MBM enforcement dates). Therefore, all possible outcomes are represented in the respective fuzzy number. The NPVs in year 10 for each scenario are to be used for the FPOM. It can be seen that the PAYS model lowers the average deviation from the base case compared to the purchase scenario, though the applied MBM enforcement date and carbon pricing sensitivities are not material enough to make the Pur_worst_ scenario less attractive than the PAYS_worst_ scenario, which would also offer protection against other risks such as low fuel prices, which are not stressed in the above. Further, favourable circumstances such as high carbon prices and an earlier than expected MBM enforcement cannot be fully utilized under the PAYS scenario. In order to compare both financing methods not only visually but also numerically, the fuzzy numbers have to be converted into the respective FNPV:

7 $${\mathrm{FNPV}}_{\mathrm{Purchase}}=E({A}_{+})\times \frac{{\int }_{0}^{\infty }{\mu }_{A}(\mathrm{NPV})\mathrm{dNPV}}{{\int }_{-\infty }^{\infty }{\mu }_{A}(\mathrm{NPV})\mathrm{dNPV}}$$

8 $$=(a+\frac{\beta -\alpha }{6})\times \frac{0.5\times (\alpha +\beta )}{0.5\times (\alpha +\beta )}$$

9 $$=\mathrm{1,377.86}\times 1=\mathrm{1,377.86} \mathrm{\:USDk}$$

10 $${\mathrm{FNPV}}_{\mathrm{PAYS}}=E({A}_{+})\times \frac{{\int }_{0}^{\infty }{\mu }_{A}(\mathrm{NPV})\mathrm{dNPV}}{{\int }_{-\infty }^{\infty }{\mu }_{A}(\mathrm{NPV})\mathrm{dNPV}}$$

11 $$=(a+\frac{\beta -\alpha }{6})\times \frac{0.5\times (\alpha +\beta )}{0.5\times (\alpha +\beta )}$$

12 $$=\mathrm{1,301.82}\times 1=\mathrm{1,301.82} \mathrm{\:USDk}$$

Please refer to the literature review section for the definition of a, b, α and β (Fig. 1). When looking at the VLSFO case, FNPV_Purchase (VLSFO)_ equals USDk 1,809.51 and FNPV_PAYS (VLSFO)_ equals USDk 1,528.66.

Now, as the risks and opportunities linked to MBMs for a Flettner Rotor are boiled down to one number, the FNPV, the shipping company can use it along with other KPIs such as the IRR, which would reflect the lower upfront payment when using PAYS, to compare the technology with other abatement options and financing options.

It needs to be said that the scenarios above neglected the impact of volatile fuel prices in order to highlight the carbon pricing (i.e. MBM) sensitivity. However, the literature review shows that fuel prices have a significant impact on the returns of the respective greening technology. In Appendix 2, the interplay of carbon and fuel prices (both IFO380 and VLSFO scenario) is shown in tables, which are coloured based on a normal distribution. The tables confirm that the fuel prices have a higher impact on the returns than carbon pricing, which has already been highlighted in the literature review. However, the table also shows that carbon pricing would provide a security buffer in low fuel price (i.e. < ( −)40%) scenarios. For example, in a ( −)50% fuel price scenario, carbon pricing of USD 30–40 per ton would compensate enough losses to set the NPV of the investment to zero for both scenarios (IFO380 and VLSFO).

## Interpretation of results

The results indicate that investments in greening technologies do not necessarily require MBMs in order to pay off. The main challenges such as the sufficient access to the required funds may not be solved by increasing the investment’s attractiveness, as the IRRs of such investments are already promising. However, carbon prices of USD 100 per ton might increase the NPV of a technology substantially (Fig. 5) and prices such as discussed by ben Brahim et al. (2019) would have an even larger impact. The applied PAYS model (Schinas and Metzger 2019a), which de-risks a technology investment for a shipping company, addresses the financing problem.

The numerical example does not consider subsidies or other financial support (i.e. export credit schemes). However, in some of the MBM proposals, the paid levies flow back to the shipping industry in order to support the installation of greening technologies. Such double incentives would decrease the financing costs for cost-intensive greening technologies and thereby enhance the return profile of such a technology.

Looking at the WASP technologies, they might be a promising abatement option (Rehmatulla et al. (2017) come to the same conclusion) but require substantial cash commitment. Alternative financing models such as PAYS reduce the required funds for such a technology but require a counterparty that is willing to take the risks connected to the investment. However, as the example indicates, MBMs are not necessarily required for a rewarding investment, though they would materially increase its attractiveness.

The analysis considered IFO380 and VLSFO, while the latter is 25% more expensive and 4.5% more efficient. Further, in order to make both fuels comparable, another 5% efficiency premium has been considered since the ship that uses IFO380 most likely uses a scrubber. The results suggest that, from a pure economical perspective, a WASP investment makes even more sense when using VLSFO since the efficiency increase does not fully compensate the higher price when compared to IFO380. This is also reflected in the respective FNPVs.

The author aimed to make the numerical example as realistic as possible. Anyhow, it needs to be mentioned that there are limitations. The tables in Appendix 2 show that the impact of fuel prices can be more critical for a technology investment than carbon prices (as long as the latter stay in the range of the presented scenarios), although there is no doubt that carbon pricing improves the IRR of any carbon emission reduction technology and contributes to its NPV. Further, it can be argued that carbon offset revenues provide an additional layer of security in unfavourable low fuel price scenarios.

Other notable limitations are.

- the performance of WASP technologies inter alia depends on ship, routes and weather, which is why the actual performance over the lifetime needs to be assessed on a case-by-case basis;
- the focus on WASP technologies (other technologies (see Bouman et al. (2017)) also have promising carbon offset potential);
- the total carbon footprint of WASP technologies (i.e. production and disposal) is barely researched and therefore neglected in the conducted analysis, though this becomes relevant when preparing a holistic ecological assessment. It needs to be noted that the challenge in relation to the “second life” of the technology (i.e. recycling, repowering etc.) has so far been pushed into the future, which is not satisfying from a research perspective.

Finally, it can be said that the FPOM is a feasible and easy-to-use method for assessing the chances and opportunities linked to technological measures (see also Metzger and Schinas (2019)). The methodology can be extended by other financing schemes (i.e. leasing) and uncertainties (i.e. fuel prices), which would enhance its relevance for the decision-making process. In order to experience the full value provided by the FPOM to the decision-making process, multiple abatement options have to be evaluated (visually and numerically). This might be interesting for further research.

## Conclusion

The aim of this paper is to assess the impact of MBMs on greening technologies. Therefore, a literature review on MBMs and green shipping was conducted first. Afterwards, a numerical example, which considers a plain vanilla purchase of a technology as well as the PAYS model, is used to visualize the impact of MBMs on greening technologies. Figure 9 summarizes the results for a Flettner Rotor investment by applying the FPOM.

In the introduction, the question whether MBMs are necessary to make investments in greening technologies (and especially WASP technologies) attractive is raised. This paper shows that greening technologies are also attractive without MBMs from an economic point of view. However, carbon prices can have a significant impact on the returns and can provide an additional layer of security in extreme scenarios (i.e. drop of fuel prices, see Appendix 2). The analysis assumes that greening technologies, which are installed in the year 2021, will most likely benefit from an enforced MBM in c. 5 years. The applied PAYS model mitigates the impact of such uncertainties on the NPV, though it also limits the utilization of favourable circumstances such as an earlier than expected MBM enforcement.

Further, the analysis suggests that the economic profile of a greening technology becomes even more attractive when the ship uses VLSFO rather than IFO380 in combination with a scrubber.

The applied methodology can be used to numerically and visually compare multiple greening technologies (i.e. detachable sails or alternative fuels) and associated financing schemes with each other, which also makes it relevant for other research efforts in the field of green ship finance.

## Figures and Tables

**Fig. 10:**
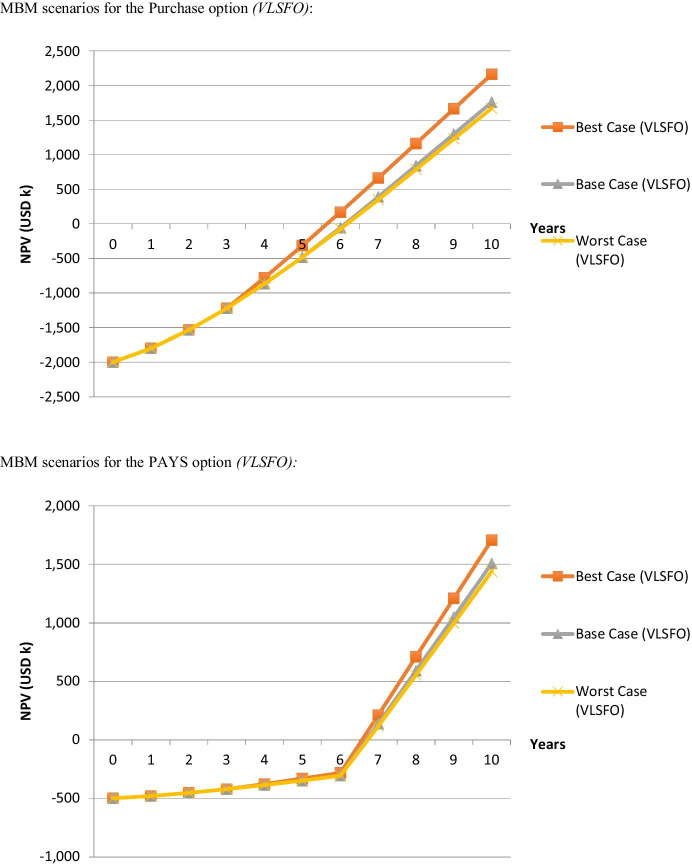
VLSFO scenarios

**Fig. 11:**
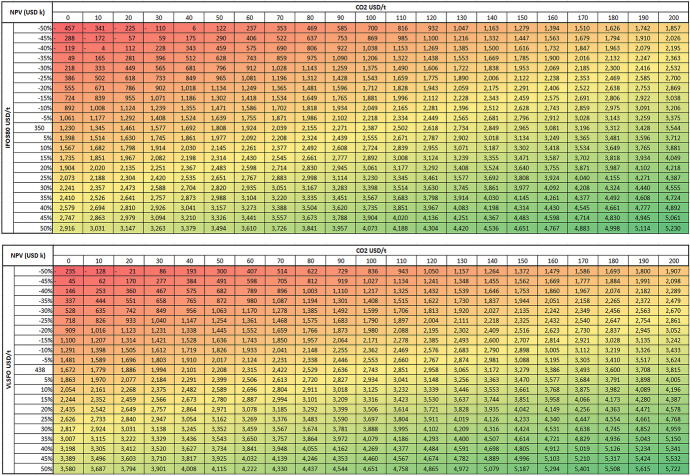
Carbon and fuel price sensitivity

## Data Availability

Figures 1 and 2 are copied from Metzger and Schinas (2019).

## Appendix 1

Figure 10

## Appendix 2

Figure 11
